# PCI-24781, a Novel Hydroxamic Acid HDAC Inhibitor, Exerts Cytotoxicity and Histone Alterations via Caspase-8 and FADD in Leukemia Cells

**DOI:** 10.1155/2010/207420

**Published:** 2010-01-18

**Authors:** Nilsa Rivera-Del Valle, Shan Gao, Claudia P. Miller, Joy Fulbright, Carolina Gonzales, Mint Sirisawad, Susanne Steggerda, Jennifer Wheler, Sriram Balasubramanian, Joya Chandra

**Affiliations:** ^1^Department of Pediatrics Research, Children's Cancer Hospital, The University of Texas M. D. Anderson Cancer Center, Houston, TX 77030, USA; ^2^Graduate School of Biomedical Sciences, The University of Texas Health Science Center at Houston, Houston, TX 77030, USA; ^3^Pharmacyclics Inc., Sunnyvale, CA 94085, USA; ^4^Department of Investigational Therapeutics, The University of Texas M. D. Anderson Children's Cancer Hospital, Houston, TX 77030, USA

## Abstract

Histone deacetylase inhibitors (HDACi) have become a promising new avenue for cancer therapy, and many are currently in Phase I/II clinical trials for various tumor types. In the present study, we show that apoptosis induction and histone alterations by PCI-24781, a novel hydroxamic acid-based HDAC inhibitor, require caspase-8 and the adaptor molecule, Fas-associated death domain (FADD), in acute leukemia cells. PCI-24781 treatment also causes an increase in superoxide levels, which has been reported for other HDACi. However, an antioxidant does not reverse histone alterations caused by PCI-24781, indicating that ROS generation is likely downstream of the effects that PCI-24781 exerts on histone H3. Taken together, these results provide insight into the mechanism of apoptosis induction by PCI-24781 in leukemia by highlighting the roles of caspase-8, FADD and increased superoxide levels.

## 1. Introduction

Epigenetics is currently defined as the heritable changes in gene expression without alterations in DNA sequence [1]. Epigenetic alterations include histone modification, DNA methylation, and microRNA expression. In particular, abnormal histone tail modifications such as acetylation have been linked to tumor progression [2]. Histone acetylation is modulated by two families of enzymes: histone deacetylases (HDACs) and histone acetyltransferase (HATs). Irregular patterns of histone acetylation have been hypothesized to silence tumor suppressor genes in human cancer cells [3]. Consequently, restoring the normal complement of gene expression has become a therapeutic goal. The HDAC inhibitors (HDACi) are a structurally diverse family of anticancer drugs that target these abnormal histone acetylations by inhibiting HDAC enzymes [4]. In mammalian systems, eleven HDAC enzymes are grouped into four classes based on structural and functional characteristics. Consequently, the HDACi compounds are often categorized based on their ability to inhibit various HDAC classes. The approval of vorinostat (suberoylanilide hydroxamic acid (SAHA)), a pan-HDAC inhibitor, by the U.S. Food and Drug Administration for treatment of cutaneous T-cell lymphoma [5] was a recent major milestone in validating the clinical utility of this class of compounds. This success has encouraged the preclinical and clinical developments of dozens of other HDACi. 

One such compound is PCI-24781 (formerly known as CRA-024781), a novel, orally dosed HDACi. Like vorinostat, PCI-24781 is a hydroxamic acid, and can inhibit all Class I and Class II HDAC isoforms, although it is reported to be a more potent inhibitor of HDACs 1 and 3 at low concentrations [6]. Assessment of in vitro activity against tumor cell lines revealed growth inhibition of multiple solid tumor lines including colon, breast, lung, prostate, ovarian, Hodgkins lymphoma, and non-Hodgkins lymphoma [7]. Only one published study has probed the mechanism of cell death induced by PCI-24781 in a series of lymphoma lines and reported caspase activation and generation of reactive oxygen species, consistent with the mechanism of cytoxicity of other HDACi [7]. Tumor inhibition and histone acetylation were also noted *in vivo *in glioma, colon, and lung tumor xenograft models [6]. 

Our current study seeks to extend these mechanistic studies to acute leukemia cells and to clarify the specific role of caspase-8 and the adaptor molecule Fas-associated death domain (FADD) in the mechanism of apoptosis induced by PCI-24781. Effects on acetylation of histone H3 by PCI-24781 were also examined in acute lymphocytic leukemia (ALL) cells and in variants lacking caspase-8 or FADD, and revealed a lower degree of histone H3 acetylation in the latter lines. This surprising result highlights the importance of these two components of the Fas receptor pathway in conferring sensitivity to PCI-24781 in acute lymphocytic leukemia cells. 

## 2. Material and Methods

### 2.1. Cell Lines

Jurkat, I2.1 (FADD deficient Jurkat cells) and CEM human leukemia cell lines were acquired from American Type Culture Collection (Manassas, VA). I9.2 (caspase-8 deficient Jurkat cells) were provided by Dr. Michael Andreeff (The University of Texas M. D. Anderson Cancer Center (UTMDACC), Houston, TX). All cells were grown in a humidified incubator with 5% CO_2_ at 37°C and cultured in RPMI 1640 with 10% (v/v) heat-inactivation fetal bovine serum (Hyclone, Logan, UT), 2 mM L-glutamine, 100 U/mL penicillin, and 100 *μ*g/mL streptomycin (Sigma St. Louis, MO).

### 2.2. Reagents

PCI-24781 was kindly provided by Pharmacyclics Inc. (Sunnydale, CA). Trypsin-ethylenediaminetetraacetic acid (EDTA), propidium iodide (PI), N-acetyl cysteine (NAC), Buthionine sulfoximine (BSO), and Triton X-100 were purchased from Sigma (St. Louis, MO). Dye for the detection of intracellular superoxide (dihydroethidium [HEt]) was purchased from Molecular Probes (Eugene, OR). Caspase-3 substrate, DEVD-amc, was purchased from Biomol International, LP (Plymouth Meeting, PA). The caspase inhibitors zVAD-fmk and IETD-fmk were purchased from Calbiochem (San Diego, CA). Antibodies were purchased for caspase-3 (Cell Signaling, San Diego, CA), polyclonal anti-acetyl-histone H3 (Abcam, Inc., Cambridge, MA), and actin (Sigma). Annexin V-fluorescein isothiocyanate (Annexin V-FITC) was purchased from BD Bioscience (Franklin Lakes, NJ). QVD-OPH was purchased from MBL International (Woburn, MA).

### 2.3. Assessment of DNA Fragmentation

Apoptosis was assessed by determining the percentage of subdiploid cells using PI staining followed by flow cytometric analysis as previously described [8]. The cells were incubated for 24 hours, centrifuged, and resuspended in 500 *μ*L of PI solution (50 *μ*g/mL PI, 0.1% Triton X-100, and 0.1% sodium citrate in PBS). Samples were assessed by flow cytometry on the FL-3 channel (FACSCalibur, Becton, Dickinson, Franklin Lakes NJ). CellQuest software was used for the analysis of the data (BD Bioscience, Franklin Lakes NJ).

### 2.4. Annexin V Staining

Phosphatidylserine was measured by Annexin V-FITC staining according to the manufacturer's protocol. CEM cells were pretreated with 5 *μ*M QVD-OPH (a pan caspase inhibitor) and treated with 0.5 *μ*M PCI-24781 for 30 hours, washed twice in cold PBS, resuspended in 1 X binding buffer (0.01 M HEPES, pH 7.4; 0.14 M NaCl; 2.5 mM CaCl_2_), and incubated for 30 minutes in the dark at room temperature with 5 *μ*L Annexin V-FITC and 10 *μ*L of 50 *μ*g/mL PI. Samples were analyzed by flow cytometry on the FL-1 (for FITC) and FL-3 (for PI) channels and analyzed using CellQuest software.

### 2.5. Detection of Intracellular Superoxide

The intracellular superoxide level was measured using the cell-permeable HEt dye as previously described [9]. Cells were centrifuged and resuspended in 1 mL of phosphate buffered saline (PBS) containing 10 *μ*M HEt. The samples were incubated for 30 minutes in the dark at 37°C. Fluorescence intensity was assessed by flow cytometer on the FL-3 (for HEt) channel and analyzed by CellQuest software. 

### 2.6. Caspase-3-Like Activity Assays

Cells were centrifuged, resuspended in 100 *μ*L PBS and lysed by freezing and thawing. To each well, 50 *μ*L of lysate and 150 *μ*L of 50 *μ*M DEVD-amc in DEVD buffer (10% sucrose, 0.001% IGEPAL, 0.1% CHAPS, 5 mM HEPES, pH 7.25) were added in duplicates on a 96-well plate. The release of fluorescence (amc) generated from the cleavage of DEVD-amc was measured using a spectrofluorometer (SpectraMax Gemini EM, Molecular Devices, Sunnyvale, CA) using an excitation of 355 nm and emission of 460 nm.

### 2.7. Western Blotting

After treatment, Jurkat cells (5 × 10^6^) were resuspended in lysis buffer (1% Triton X-100, 150 mM NaCl, 5 mM EDTA, 20 mM sodium phosphate, pH 7.4). Aliquots of protein lysates (30 *μ*g) were loaded on 12% sodium dodecyl sulfate- (SDS) polyacrylamide gels, transferred to nitrocellulose membranes, and blocked overnight at 4°C with 5% nonfat dry milk in Tris-Buffered Saline 0.05% Tween-20 (TBS-T). Membranes were probed with 1 : 1000 dilution of primary antibody in 5% milk in TBS-T. The bound antibodies were detected using enhanced chemiluminescence, ECL plus Western blotting detection system (Amerham Bioscience, UK limited, Little Chalfont Buckinghamshire, England).

### 2.8. Statistical Analysis

For each condition, multiple experiments were performed, and the results are presented as the mean ± standard deviation (S.D). The differences between two group conditions were analyzed using independent, two-tailed *t* *-*tests (Microsoft Excel software, Redmond, WA).

## 3. Results

### 3.1. PCI-24781 Induces In Vitro Apoptotic Cell Death in Leukemia Cells

**Figure 1:**
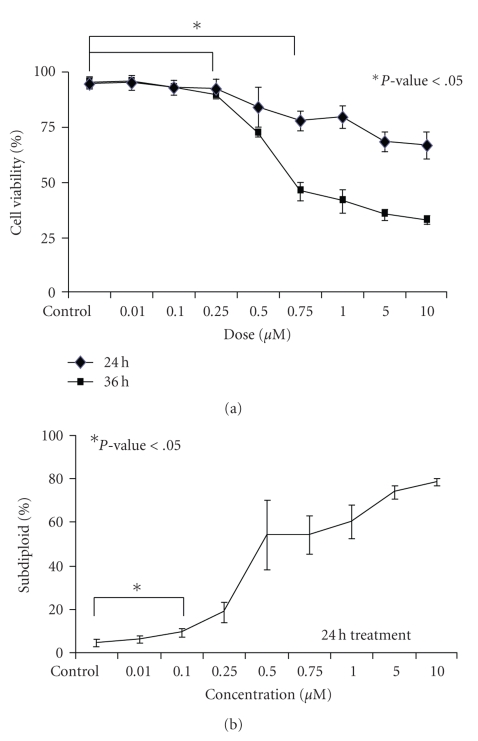
*The exposure of PCI-24781 increases cytotoxic effects of leukemic cells*. Jurkat cells were treated with a range of PCI-24781 doses (0.01 *μ*M–10 *μ*M), (a) incubated for 24 and 36 hours, and the % cell viability was quantitated by trypan blue exclusion using a Vi-Cell Coulter Counter, (b) incubated for 24 hours, dyed with PI reagent, and analyzed by flow cytometry to assess DNA fragmentation. Error bars represent the means ± S.D. of three independent experiments. *P < .05 compared to control.

Previous research indicated that PCI-24781 is cytotoxic in multiple solid tumor lines [6], however the study of this drug in hematopoetic cells is limited to one study conducted in Hodgkins lymphoma and non-Hodgkins lymphoma cell lines [7]. To extend these mechanistic studies to leukemia cells, the cytotoxic effects of PCI-24781 were investigated in an ALL cell line. Jurkat cells were treated with a range of PCI-24781 doses (0.01 *μ*M–10 *μ*M), incubated for 24 hours and 36 hours, and percent viability was quantified by trypan blue exclusion. As seen in Figure 1(a), there was a significant reduction in Jurkat cell viability beginning at the 0.25 *μ*M and 0.75 *μ*M dose of PCI-24781 after exposure for 24 or 36 hours, respectively, (*P-*value < .05).

Having identified doses at which PCI-24781 is cytotoxic to ALL cells, the next step was to examine whether the observed cell death was due to apoptosis. DNA fragmentation is a well-defined characteristic of apoptosis and can be quantified by measuring the increase in the percentage of cells containing subdiploid amounts of DNA by staining cells with PI. Jurkat cells were treated with a range of PCI-24781 doses (0.01 *μ*M–10 *μ*M), incubated for 24 hours, stained with PI and assessed by flow cytometry. Figure 1(b) shows that a 24-hour exposure to PCI-24781 led to a dose-dependent increase in DNA fragmentation beginning at the 0.1 *μ*M dose (*P-*value < .05).

### 3.2. PCI-24781 Induced Apoptosis Is Caspase Dependent

**Figure 2:**
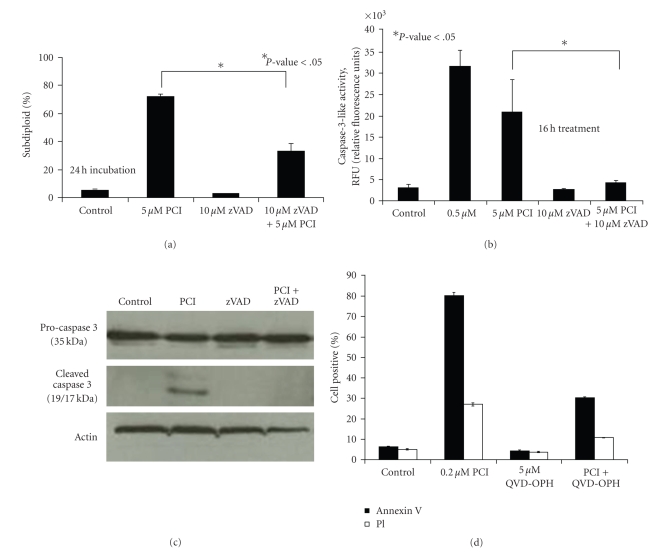
*The exposure of PCI-24781 induces caspase-dependent apoptosis in leukemic cells*. (a) DNA fragmentation was assessed in Jurkat cells treated with 5 *μ*M of PCI-24781 (PCI), with or without 30 minutes of zVAD-fmk pretreatment, incubated for 24 hours, dyed with PI reagent, and assessed on the flow cytometer. (b) *Jurkat* cells were treated with 0.5 *μ*M and 5 *μ*M PCI-24781, with or without 30 minutes of zVAD-fmk pretreatment, and incubated for 16 hours. Incubated cells were lysed and stained with DEVD-amc to measure caspase-3-like activity. The release of amc was measured on a spectrofluorometer using an excitation of 355 nm and an emission of 460 nm. (c) In protein lysates from cells treated with 5 *μ*M PCI-24781 for 16 hours, procaspase 3, cleaved caspase 3, and actin were measured by western blot. (d) CEM cells were pretreated with 5 *μ*M QVD-OPH for 2 hours and treated with 0.5 *μ*M PCI-24781 for 30 hours. Percentage cells positive with Annexin V/PI staining was measured for both. Error bars ((a), (b), and (d)) represent the means ± S.D. of three independent experiments. ((a) and (b)) *P < .05 relative to 5 *μ*M PCI-24781.

Having demonstrated that the cytotoxic effects of PCI-24781 in ALL cells involve DNA fragmentation, we next investigated if a caspase-dependent apoptotic pathway was activated. Jurkat cells were pretreated with 10 *μ*M zVAD-fmk (a pan caspase inhibitor) for 30 minutes and then treated with 5 *μ*M PCI-24781 for 24 hours, followed by PI staining and flow cytometry. As shown in Figure 2(a), the pan-caspase inhibitor alone had no effect on DNA fragmentation. However, apoptotic DNA fragmentation induced by PCI-24781 was significantly reduced when caspase activity was blocked (*P-*value < .05).

**Figure 3:**
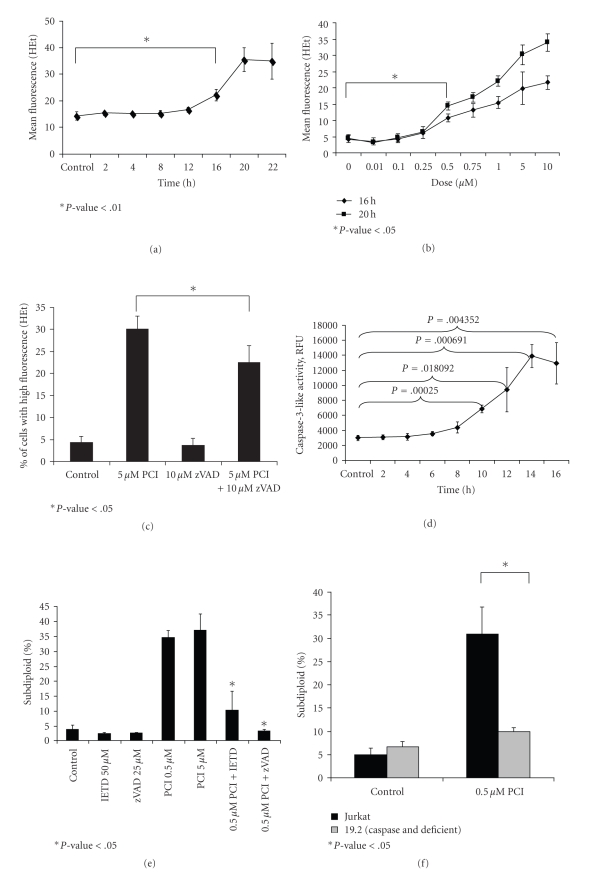

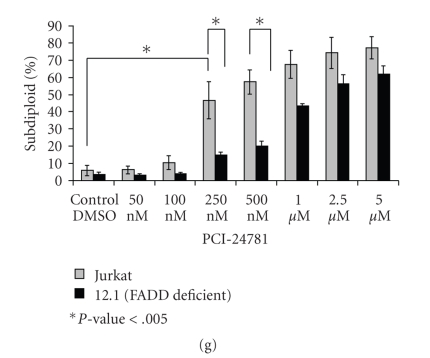
*PCI-24781 induces ROS generation in a caspase-dependent and time-dependent manner*. (a) Jurkat cells were treated with 5 *μ*M of PCI-24781, incubated between 2 and 22 hours. Intracellular superoxide was measured at each time point using dihydroethidium (HEt) and assessed by flow cytometry. *P < .01 compared to control. (b) Jurkat cells were treated with a range of PCI-24781 doses (0.01 *μ*M–10 *μ*M), and incubated 16 and 20 hours. The mean HEt fluorescence at each dose was measured by flow cytometry. *P < .05 compared to control. (c) Jurkat cells were treated with 5 *μ*M PCI-24781, with or without zVAD-fmk pretreatment, and incubated for 24 hours, intracellular superoxide levels were assessed using HEt. *P < .05. (d) Jurkat cells were treated with 5 *μ*M PCI-24781, incubated between 4 and 16 hours, lysed and stained with DEVD-amc to assess caspase-3-like activity. Caspase-3 activity was measured in relative fluorescence unit (RFU). The release of amc was measured on a spectrofluorometer using an excitation of 355 nm and an emission of 460 nm. *P < .05 compared to control. (e) Jurkat cells were pretreated with either inhibitor of caspase 8 (IETD-fmk) or a pan caspase inhibitor (zVAD-fmk) for 30 minutes. After pretreatment, the samples were treated with 0.5 or 5 *μ*M PCI-24781 for 16 hours and dyed with PI reagent. DNA fragmentation (% subdiploid) was assessed by flow cytometer. (f) Jurkat and I9.2 cells were treated with or without 0.5 *μ*M PCI-24781 and incubated by 16 hours, dyed with PI reagent, and assessed on the flow cytometer. *P < .05 relative to Jurkat 0.5 *μ*M PCI-24781. (a)–(f) Error bars represent the means ± S.D. of three independent experiments. (g) Jurkat and I2.1 cells were treated with or without 0.5 or 5 *μ*M PCI-24781 and incubated by 16 hours, dyed with PI reagent, and assessed on the flow cytometer. *P < .05 relative to Jurkat cells treated with PCI-24781.

Since caspase-3 activation induces apoptotic DNA fragmentation, this end point was specifically examined in Jurkat cells in response to treatment with PCI-24781. Caspase-3-like activity was measured by monitoring fluorescence levels generated from the hydrolysis of the DEVD-amc fluorogenic substrate. Jurkat cells were pretreated with zVAD-fmk for 30 minutes, and then treated with 5 *μ*M PCI-24781 for 16 hours. Figure 2(b) shows that 5 *μ*M PCI-24781 increased caspase-3-like activity by 7-fold as compared with control. In addition, pretreatment with the pan caspase inhibitor, zVAD-fmk, successfully abrogated the increase of caspase-3-like activity induced by 5 *μ*M PCI-24781 (*P*-value < .05). Although caspase-3-like activity was higher with the 0.5 *μ*M dose compared to 5 *μ*M PCI-24781, these results most likely reflect that the higher dose (5 *μ*M) is peaking at an early time point. This idea is supported by Figure 3(d), in which a time course with 5 *μ*M revealed that maximum levels are reached at 14 hours and begin to decline after this time point. Analysis of later time points, after 16 hours, most likely will further support this idea. 

DEVD-amc has been criticized as a nonspecific substrate for caspase-3, because it can detect caspase-3 and/or caspase-7 activities. Caspase activation can also be measured by western blotting to visualize the cleavage of the large and small subunits of the caspase. To investigate if PCI-24781 specifically results in caspase-3 activation, cleaved caspase-3 was measured by western blot. The 19-kDa and 17-kDa cleaved products were evident after treatment with 5 *μ*M PCI-24781, but there was no caspase-3 cleavage when the drug was combined with zVAD-fmk pretreatment (Figure 2(c)), verifying that caspase-3 activation is a consequence of PCI-24781 treatment.

In order to further validate the results in Jurkat cells, apoptosis was measured in a different ALL cell line (CEM) and by detection of a different biochemical event that occurs during apoptotic cell death. Annexin V binds to phosphatidylserine displayed on the cell membrane, which is required for efficient disposal of the apoptotic cell. CEM cells were pretreated with 5 *μ*M QVD-OPH and treated with 0.2 *μ*M PCI-24781 for 30 hours. Cells were stained with Annexin V/PI and then analyzed by flow cytometry. As expected, in CEM cells, the percentage of Annexin V positive cells increases with PCI-24781 treatment and decreases when caspase activation is inhibited in PCI-24781 treated cells (Figure 2(d)).

### 3.3. PCI-24781 Induces ROS Generation in a Caspase-Dependent and Time-Dependent Manner

ROS have been shown to induce apoptosis by the release of cytochrome c from the mitochondria, which activates the caspase cascade. Previous studies have shown that many cancer cells have higher ROS levels compared to normal cells [10]. Therefore, one therapeutic approach is to use ROS generating anticancer agents that push intracellular ROS levels beyond a critical threshold and induce apoptosis. Various anticancer drugs like HDAC inhibitors have been shown to increase intracellular ROS levels as a single agent [11]. Since we observed caspase-3 activation in ALL cells treated with PCI-24781, we sought to determine the role of ROS by measuring intracellular superoxide levels. Jurkat cells were treated with 5 *μ*M of PCI-24781, incubated for various times spanning 2–22 hours, stained with dihydroethidium, and analyzed by flow cytometry. As shown in Figure 3(a), ROS increased in a time-dependent manner beginning at 16 hours (*P-*value < .01) and peaks at 20 hours. Figure 3(b) shows that ROS also increases in a dose-dependent manner with PCI-24781 treatment, beginning at 0.5 *μ*M at both 16 hours and 20 hours time points (*P*-value < .05).

Thus, so far our findings indicate that PCI-24781 induces apoptosis, which can be linked to ROS generation and/or caspase activation. The next step was to determine if ROS generation precedes or follows caspase activation. Superoxide levels were measured after exposure to 5 *μ*M PCI-24781, with or without pretreatment of zVAD-fmk. Figure 3(c) shows that when caspase activity is blocked, PCI-24781 induced ROS generation is blunted (*P-*value = .03). Therefore, caspase activation plays a role in the increase of ROS levels seen with PCI-24781 treatment. Next, the kinetics of caspase activation was examined. Jurkat cells treated with 5 *μ*M PCI-24781 were incubated between 4 and 16 hours, and caspase-3-like activity was measured using DEVD-amc as a substrate (Figure 3(d)). Since no ROS generation was observed at 8 hours of exposure to the same dose of PCI-24781 (data not shown), these results demonstrate that caspase activation occurs first, followed by ROS generation.

A single study has examined the molecular mechanism of apoptosis induction by PCI-24781 and reported that both caspase-8 and -9 are cleaved and activated by the HDACi in lymphoma lines. In order to more carefully examine the role of caspase-8 in PCI-24781 induced cell death, we used various peptide-based caspase inhibitors. Jurkat cells were treated with 0.5 *μ*M and 5 *μ*M PCI-24781, with or without pretreatment with either zVAD-fmk (a pan caspase inhibitor) or an inhibitor of caspase-8 (IETD-fmk). After 16 hours the cells were stained with PI reagent and DNA fragmentation was assessed by flow cytometer as shown in Figure 3(e). Results show no significant difference between 0.5 *μ*M and 5 *μ*M doses. Since 0.5 *μ*M represents a potentially less toxic and more clinically relevant dose, we decided to combine the lower dose (0.5 *μ*M) with the caspase inhibitors. DNA fragmentation by PCI-24781 was significantly reduced in the presence of IETD-fmk, suggesting that caspase-8 is involved in the induction of apoptosis by PCI-24781. To validate this result, I9.2 cells (a caspase-8 deficient Jurkat variant) [12] were treated with 0.5 *μ*M PCI-24781 for 16 hours. Figure 3(f) shows a significant decrease of DNA fragmentation in I9.2 cells treated with the HDACi as compared with wildtype Jurkat cells, demonstrating again the importance of caspase-8 in PCI-24781 induction of apoptosis. Activation of caspase-8 requires participation of an adaptor molecule which links the death receptor (Fas in this case) with caspase activation. A second Jurkat cell variant, this one lacking FADD (I2.1), was used to further probe the functionality of the Fas pathway [12]. Figure 3(g) indicates that FADD deficiency reduces DNA fragmentation by PCI-24781.

### 3.4. PCI-24781 Induces Total and Acetylated H3 Protein Expression in a Caspase and FADD Dependent Manner

**Figure 4:**
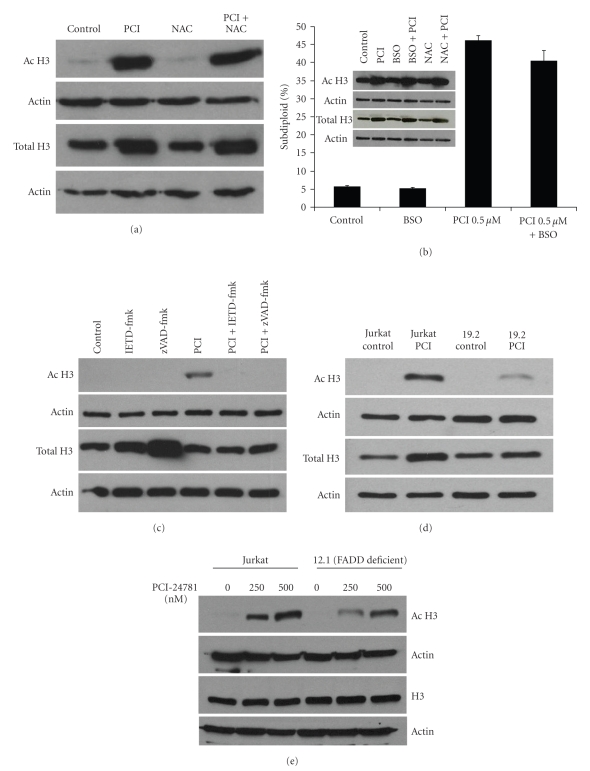
*PCI-24781 induces a caspase-dependent increase in both total and acetylated histone H3 protein expressions*. Jurkat cells were treated with 0.5 *μ*M of PCI-24781, with or without pretreatment of (a) 24 mM NAC, (b) 1 mM buthionine sulfoximine (BSO), (c) 50 *μ*M zVAD-fmk or IETD-fmk, for 30 minutes. After 16 hours of incubation, samples were either harvested to determine DNA fragmentation by PI staining or lysed for western blot. Acetylated histone H3, total histone H3, and actin were measured by western blot. (d) Jurkat and I9.2 cells were treated with or without 0.5 *μ*M PCI-24781 and incubated for 16 hours. Lysates were harvested and lysed. Acetylated histone H3, total histone H3, and actin were measured by western blot. (e) Jurkat and I2.1 cells were treated with or without 0.25 or 0.5 *μ*M PCI-24781 and incubated for 16 hours. Samples were harvested and lysed. Acetylated histone H3, total histone H3, and actin were measured by western blot. For Figures 4(a)–4(d), results are representative of at least two independent experiments.

The primary mechanism of action of HDAC inhibitors is to prevent abnormal deacetylation of histones by antagonizing HDAC enzymes. This mechanism has been hypothesized to increase expression of tumor-suppressor genes. In order to test whether PCI-24781 induced histone acetylation is linked to the apoptotic pathway and generation of ROS, Jurkat cells were treated with 0.5 *μ*M of PCI-24781, with or without pretreatment of NAC (an antioxidant that works by increasing levels of the most abundant intracellular antioxidant, glutathione (GSH)), BSO (an agent that depletes GSH) zVAD-fmk (the pan-caspase inhibitor), or IETD-fmk (caspase-8 inhibitor), for 30 minutes. After 16 hours of incubation, acetylated histone H3 (Ac H3) and total histone H3 (total H3) were measured by western blot. In addition DNA fragmentation was assessed for BSO pretreatment samples. Results show that, as expected, PCI-24781 exposure leads to an increase in acetylated histone H3 protein levels (Figures 4(a), 4(b) and 4(c)). Neither NAC nor BSO changed the increase in Ac H3 (Figures 4(a) and 4(b)), indicating that boosting or depleting GSH, respectively, has no effect on PCI-24781's ability to hyperacetylate histone H3. Consistent with this result, BSO depletion of GSH did not promote further DNA fragmentation induction by PCI-24781 when the two compounds were combined (Figure 4(b)). In contrast, inhibition of caspase activation with IETD-fmk or zVAD-fmk blocked the increase in Ac H3 protein levels (Figure 4(c)), implicating caspases in elevated levels of acetylated histone H3. To confirm the results obtained with the caspase-8 inhibitor, IETD-fmk, we examined the effects of PCI-24781 exposure on histone H3 acetylation in I9.2 cells. Our findings indicate that there was less of an increase in Ac H3 protein levels in caspase-8 deficient I9.2 cells as compared to Jurkat cells (Figure 4(d)). Similar results were obtained using FADD deficient I2.1 cells, supporting a role for FADD in the mechanism of PCI-24781-mediated histone H3 acetylation (Figure 4(e)). The difference between I2.1 and wildtype Jurkat cells was more apparent at the lower (250 nM) dose of PCI-24781, indicating that dose escalation could overcome the effect of FADD deficiency.

## 4. Discussion

The current study focuses on the cytotoxic effects of a hydroxamic acid HDACi, PCI-24781, in leukemia cells. Using Jurkat cell variants that lack caspase-8 (I9.2) or FADD (I2.1), we show that apoptosis induction and histone acetylation by this HDACi are dependent upon these two proapoptotic molecules. In particular, the effects of FADD deficiency are suggestive of a role for the extrinsic apoptotic pathway triggered by Fas/Fas ligand interactions in these cells. Data showing that inhibition of caspase activation by zVAD-fmk or a lack of FADD or caspase-8 decreases the total and acetylated protein levels of histone H3 induced by PCI-24781 was unexpected and interesting (Figures 4(c)–4(e)). Previous work in our laboratory has shown hyperacetylation of histone H3 in caspase-8 deficient cells when treated with a different HDACi, MS/SNDX-275, indicating that PCI-24781 may be unique in this regard [13]. However, a broader array of HDACi would need to be tested in order to determine if caspase-8 dependent acetylation is a feature exclusive to PCI-24781. Interestingly, this caspase-8 and FADD dependent protection against DNA fragmentation and histone alterations, however, appears to be surmountable by increased doses of PCI-24781. As shown in Figure 3(e), both the 0.5 *μ*M and 5 *μ*M doses of PCI-24781 induce similar amounts of DNA fragmentation as assessed by the percent subdiploid population. However, in comparing the degree of protection conferred by FADD deficiency for the two doses (Figure 3(g)), it is apparent that the higher dose (5 *μ*M) of PCI-24781 is less protected than the lower (0.5 *μ*M) dose. A similar pattern is observed when histone H3 acetylation by PCI-24781 is examined in FADD deficient cells (Figure 4(d)), which further supports the relationship between apoptosis induction and histone H3 acetylation. The exact mechanism linking caspase-8 or FADD to histone H3 acetylation is currently under investigation in our lab. Both HDAC dependent and independent scenarios are being considered. For HDAC dependent molecular explanations, it has been reported that caspase cleavage of HDACs can occur [14], resulting in their inactivation and histone H3 hyperacteylation. An HDAC-independent mechanism could explain increased histone H3 acetylation and total histone H3 levels if apoptotic DNA fragmentation (mediated by caspases) was causing release of histones from DNA [15]. In this case, free histone H3 (some acetylated and some unacetylated) would be detected in Triton soluble lysates to a lesser degree when caspases are inhibited. 

Our results also address a role for oxidative stress in the mechanism of action of PCI-24781. Several structurally diverse HDACi are reported to heighten intracellular levels of superoxide and peroxide [11], and similar results were obtained for PCI-24781 by others in lymphoma lines [7] and by us in leukemia lines (Figures 3(a)–3(c)). This oxidative stress appears caspase dependent since zVAD-fmk has a statistically significant, albeit modest effect (Figure 3(c)). However, the antioxidant, NAC, which effectively blunts ROS production by PCI-24781 (data not shown) did not alter histone H3 acetylation by PCI-24781 (Figure 4(a)). Since NAC possesses potent antiapoptotic effects, this result supports the notion that the histone H3 effects observed in the caspase-8 deficient and FADD deficient results are due to an HDAC dependent effect rather than a generalized apoptosis-mediated effect. Given reports from other groups that caspases can cleave specific HDAC family members rendering them inactive [14], and our results indicating that a pan-caspase inhibitor as well as a caspase-8 specific inhibitor can reverse acetylation changes by PCI-24781 (Figure 4(b)), this possibility will be further explored. The most widely cited mechanism of action for NAC's antioxidant effects is by bolstering total cellular GSH levels. We used BSO, a chemical inhibitor of the pathway critical for GSH synthesis, to determine if depletion of GSH would promote apoptosis and histone H3 acetylation by PCI-24781. It did not (Figure 4(b)), and together with the lack of effect of NAC on PCI-induced hyperacetylation, indicate that modulating GSH levels does not alter the drug's acetylation effects. 

A role for death receptor induced apoptotic pathways involving TRAIL and Fas has been investigated in the mechanism of action of HDACi other than PCI-24781 such as trichostatin A [16], MS/SNDX-275 [17], valproic acid [18], and vorinostat alone and in combination with other agents such as proteasome inhibitors [12]. Caspase-8 activation has been described in a handful of these studies, however, components and regulators of the death inducing signaling complex (DISC) which ultimately result in caspase-8 activation have only been addressed in two HDAC related papers. One study proposes that depsipeptide, (also called FR901228) can upregulate FasL at the mRNA level in osteosarcoma cells resulting in caspase-8 and -3 activation [19]. The same investigators also reported in a subsequent paper that in Fas resistant osteosarcoma cells, depsipeptide causes downregulation of c-FLIP [20]. Since c-FLIP confers resistance to Fas-mediated apoptosis, lowering levels of c-FLIP is able to overcome resistance and promote caspase-8 activation. Whether these mechanisms will hold true for PCI-24781's effects in leukemia cells remains to be determined. 

Despite the plethora of studies (including ours) citing caspase activation as a conserved event during HDACi induced cell death, numerous reports describe autophagic cell death as a consequence of treatment with this class of compounds. No study has as yet looked at PCI-24781's ability to induce auutophagy. However, a recent paper examined the caspase-8 and caspase-9 dependence of two hydroxamic acid HDACi: LAQ824 and LBH589 [21]. These investigators used a genetically tractable *in vivo* myc driven lymphoma model in which death receptor signaling was compromised due to overexpression of CrmA, a viral caspase-8 inhibitor, or due to deficiency of TRAIL. Caspase-9 deficiency and Apaf-1 deficiency were also incorporated into lymphoma model. Interestingly, none of these approaches to block caspase activation were able to prevent cell death by the two HDACi in the long term with morphological features of autophagy emerging. A caveat of these HDACi, though, is that unlike PCI-24781, these two compounds are reported to inhibit HDAC6, an HDAC family member which appears to regulate aggresome formation and autophagy [22]. In contrast, PCI-24781 displays the greatest potency for HDAC1 with no apparent selectivity for HDAC6 as compared to the remainder of the HDAC family. 

Our work is the first to implicate the adaptor protein FADD specifically in the mechanism of action of an HDAC inhibitor. Compellingly, in addition to its function in the cytoplasm proximal to the Fas receptor, FADD is reported to be localized to the nucleus of resting cells. Using an internalization defective Fas mutant lymphoma cell line, Foger et al. found that FADD retaines its ability to translocate from nuclei to cytoplasm and suggest that a caspase-8 dependent feedback loop regulates FADD trafficking [23]. This model raises interesting possibilities regarding an endogenous role for nuclear FADD in transcriptional complexes that routinely contain HDAC family members. A molecular connection between HDACs and FADD offers insight into our novel observation that FADD deficiency is a determinant of sensitivity to PCI-24781. Thus, extrapolating our findings to other HDACi and to other cancer models may contribute to efforts to maximize the therapeutic efficacy of this interesting and versatile class of agents. 

## References

[1] Feinberg AP (2007). Phenotypic plasticity and the epigenetics of human disease. *Nature*.

[2] Hadnagy A, Beaulieu R, Balicki D (2008). Histone tail modifications and noncanonical functions of histones: perspectives in cancer epigenetics. *Molecular Cancer Therapeutics*.

[3] Egger G, Liang G, Aparicio A, Jones PA (2004). Epigenetics in human disease and prospects for epigenetic therapy. *Nature*.

[4] Xu WS, Parmigiani RB, Marks PA (2007). Histone deacetylase inhibitors: molecular mechanisms of action. *Oncogene*.

[5] Duvic M, Vu J (2007). Vorinostat: a new oral histone deacetylase inhibitor approved for cutaneous T-cell lymphoma. *Expert Opinion on Investigational Drugs*.

[6] Buggy JJ, Cao ZA, Bass KE (2006). CRA-024781: a novel synthetic inhibitor of histone deacetylase enzymes with antitumor activity in vitro and in vivo. *Molecular Cancer Therapeutics*.

[7] Bhalla S, Balasubramanian S, David K (2009). PCI-24781 induces caspase and reactive oxygen species-dependent apoptosis through NF-*κ*B mechanisms and is synergistic with bortezomib in lymphoma cells. *Clinical Cancer Research*.

[8] Chandra J, Hackbarth J, Le S (2003). Involvement of reactive oxygen species in adaphostin-induced cytotoxicity in human leukemia cells. *Blood*.

[9] Chandra J, Tracy J, Loegering D (2006). Adaphostin-induced oxidative stress overcomes BCR/ABL mutation-dependent and -independent imatinib resistance. *Blood*.

[10] Trachootham D, Alexandre J, Huang P (2009). Targeting cancer cells by ROS-mediated mechanisms: a radical therapeutic approach?. *Nature Reviews. Drug Discovery*.

[11] Chandra J (2009). Oxidative stress by targeted agents promotes cytotoxicity in hematological malignancies. *Antioxidants & Redox Signaling*.

[12] Miller CP, Ban K, Dujka ME (2007). NPI-0052, a novel proteasome inhibitor, induces caspase-8 and ROS-dependent apoptosis alone and in combination with HDAC inhibitors in leukemia cells. *Blood*.

[13] Miller CP, Rudra S, Keating MJ (2009). Caspase-8 dependent histone acetylation by a novel proteasome inhibitor, NPI-0052 : a mechanism for synergy in leukemia cells. *Blood Journal*.

[14] Scott FL, Fuchs GJ, Boyd SE (2008). Caspase-8 cleaves histone deacetylase 7 and abolishes its transcription repressor function. *Journal of Biological Chemistry*.

[15] Wu D, Ingram A, Lahti JH (2002). Apoptotic release of histones from nucleosomes. *Journal of Biological Chemistry*.

[16] Kim HR, Kim EJ, Yang SH (2006). Trichostatin A induces apoptosis in lung cancer cells via simultaneous activation of the death receptor-mediated and mitochondrial pathway?. *Experimental and Molecular Medicine*.

[17] Lucas DM, Davis ME, Parthun MR (2004). The histone deacetylase inhibitor MS-275 induces caspase-dependent apoptosis in B-cell chronic lymphocytic leukemia cells. *Leukemia*.

[18] Bokelmann I, Mahlknecht U (2008). Valproic acid sensitizes chronic lymphocytic leukemia cells to apoptosis and restores the balance between pro- and antiapoptotic proteins. *Molecular Medicine*.

[19] Imai T, Adachi S, Nishijo K (2003). FR901228 induces tumor regression associated with induction of Fas ligand and activation of Fas signaling in human osteosarcoma cells. *Oncogene*.

[20] Watanabe K, Okamoto K, Yonehara S (2005). Sensitization of osteosarcoma cells to death receptor-mediated apoptosis by HDAC inhibitors through downregulation of cellular FLIP. *Cell Death and Differentiation*.

[21] Ellis L, Bots M, Lindemann RK (2009). The histone deacetylase inhibitors LAQ824 and LBH589 do not require death receptor signaling or a functional apoptosome to mediate tumor cell death or therapeutic efficacy. *Blood*.

[22] Pandey UB, Batlevi Y, Baehrecke EH, Taylor JP (2007). HDAC6 at the intersection of autophagy, the ubiquitin-proteasome system and neurodegeneration. *Autophagy*.

[23] Foger N, Bulfone-Paus S, Chan AC, Lee K-H (2009). Subcellular compartmentalization of FADD as a new level of regulation in death receptor signaling. *FEBS Journal*.

